# Structural Characterization and Immune Activity Screening of Polysaccharides With Different Molecular Weights From Astragali Radix

**DOI:** 10.3389/fphar.2020.582091

**Published:** 2020-11-24

**Authors:** Ke Li, Y-x Cao, S-m Jiao, G-h Du, Y-g Du, X-m Qin

**Affiliations:** ^1^ Modern Research Center for Traditional Chinese Medicine, Shanxi University, Taiyuan, China; ^2^ Institute of Process Engineering, Chinese Academy of Sciences, Beijing, China; ^3^ Institute of Materia Medica, Chinese Academy of Medical Sciences & Peking Union Medical College, Beijing, China

**Keywords:** Astragalus polysaccharide, different molecular weight, immunomodulatory activity, nonspecific and specific, structure characterization

## Abstract

Saccharides are the most abundant substance with the strongest immunological activity in Astragali Radix (AR). However, systematic structure study and immunoactivity screening of polysaccharides with different molecular weights (Mw) in AR have yet to be conducted. In this study, Astragalus polysaccharides (APSs) were divided into three fragments of different Mw values, >2,000 kDa (APS-Ⅰ), about 10 kDa (APS-Ⅱ), and about 300 Da (APS-Ⅲ), by using ultrafiltration for the first time. The structural differences of the three products were determined on the basis of monosaccharide composition, FT-IR spectrum, linkage analysis, and nuclear magnetic resonance analysis. Cellular immune activity experiments *in vitro* and cyclophosphamide immunosuppression animal model experiments *in vivo* for nonspecific and specific immunoactivity screening were applied to identify the most immunogenic fragment in APSs. Linkage analysis results showed that APS-Ⅰ, APS-Ⅱ, and APS-Ⅲ have different attachment sites of monosaccharide residues. Immune screening experiments indicated that the Mw of the APSs influenced their activity, and APS-Ⅱ had the strongest immunoenhancing activity among the products. This research may serve as a reference for further study on APSs with different structures and immune activities, and as a guidance for the quality control of APSs and the development of new APS products.

## Highlights

1. Astragalus polysaccharides (APSs) were divided into three fragments with different molecular weights by using ultrafiltration.
2. APS-Ⅱ is the most immunoactive part of APSs, and its molecular weight is about 10 kDa.
3. The molecular weight of APSs can significantly influence their immune activity.
4. APSs with different molecular weights have different structures.

**GRAPHICAL ABSTRACT:**
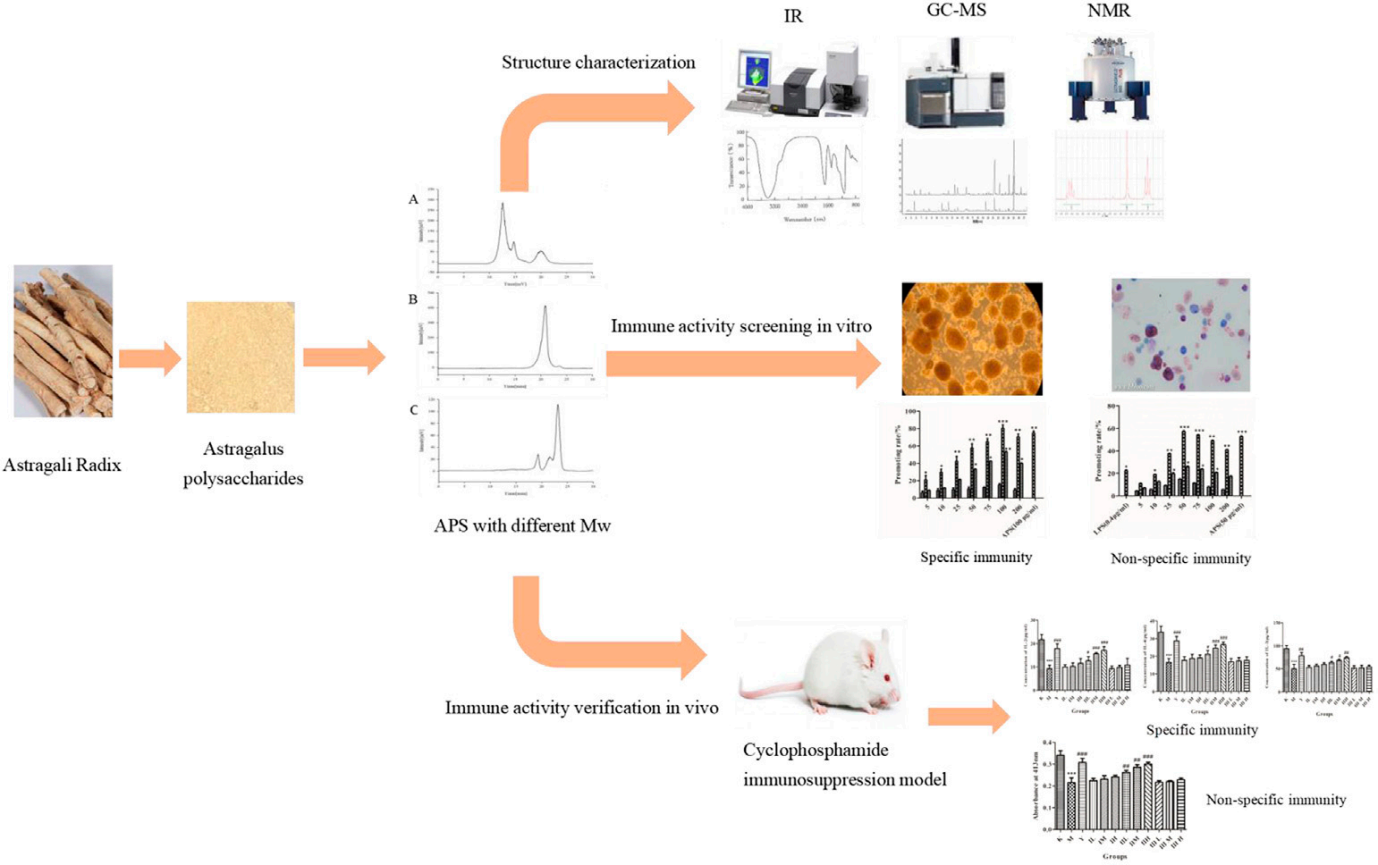
Astragalus polysaccharides with different molecular weights have different structures and effects on immune activity.

## Introduction

Astragali Radix (AR), also known as Huangqi in China, is derived from the dried root of perennial legume *Astragalus membranaceus* (Fisch.) Bge. (MJ) and *Astragalus membranaceus* (Fisch.) Bge. var. *mongholicus* (Bge.) Hsiao (MG) (Chinese Pharmacopoeia, 2015). AR has beneficial effects on the spleen and in treating *qi* deficiency and blood diseases (Sinclair et al., 1998). In clinical application, 80% prescriptions of Chinese herbal medicines contain AR. Given its importance, AR has been listed as one of the 60 strategic key varieties of the state and the 18 major varieties of Chinese medicinal materials in the Ministry of Commerce. Moreover, AR has been included in the list of national drug and food homology in 2018 (Tian et al., 2020).

AR contains many chemical components, such as saponins, flavonoids, and polysaccharides. Astragalus polysaccharides (APSs) are the most abundant substance with the strongest immunological activity in AR (Li, 2017; Zheng et al., 2020). At present, several reports are available on APSs, as shown in Table 1. The relative molecular mass distribution of APSs is very wide (5.6 × 10^3^ Da–7.6 x 10^6^ Da), which is mainly divided into dextran, arabinoxylans, and other types of polysaccharides (Liu et al., 2017; Sun et al., 2017). Moreover, APSs have a branched structure, but a method for the accurate determination of heteropolysaccharides in APSs is unavailable. The current research is mainly focused on extracting APSs from AR to study its structure and activity characteristics. However, a systematic study on the chromatographic characteristics of APS molecular weight (Mw) distribution and whether Mw affects the structure and immunomodulatory activity of APSs remains lacking.

**TABLE 1:** Astragalus polysaccharide (APS) structure characterization.

Polysaccharide name	Mw/Da	Monosaccharide composition and ratio	Structural characteristics	References
APS	7.6 × 10^6^	D-Gal、L-Ara、D-Gal A,D-Gla A (18:18:1:1)		Ai, et al. (2008)
A2Nb	3.6 × 10^5^	D-Glc	*α*-D-(1→4)-Glc	Zhu et al., (2017)
APS	3.6 × 10^5^	D-Glc	Mainly D-1,4-Glc connection, 1 out of every 25 Glc (O-6) branch	Wang et al., (2001)
Heteropolysaccharide	3.75 × 10^4^	Glc, Gal, Ara (1:0.95:0.70)	*α*-glycosidic linkage	Liu et al., (1994)
APS	2.07 × 10^4^	Glc	The main chain is repeated 1,4-Glc, connecting ten 1,6-Glc branch residues	Niu et al., (2011)
LMw-APS	5.6 × 10^3^	Glc, Gal, Ara, Gal A, Xyl (10:1.3:1.7:0.95:1)	(1→4)-Glc and a little (1→4)-Gal，(1→3)-GalA，(1→2)- Xyl	Qu et al., (2010)
APS1	2.58 × 10^5^	Glc		Jiang et al., (2016)
APS2	4 × 10^4^	Ara	—	
APS3	1.5 × 10^4^	Rha, Glc, Gal, Ara (1:10.76:6.55:12)	—	
APS4	3.2 × 10^3^	Gal, Ara (3.02:1)	—	
Water-soluble dextran （AG-1）	—	*α*-1,4 dextran, *α*-1,6 dextran	The composition ratio of *a*-1,4 and *a*-1,6 glycosidic bonds is 5:2	Huang et al., (1982)
Water-insoluble glucan （AG-2）	—	*α*-1,4 dextran	*α*-1,4 glucan linkage	
Arabinoxylan	1.04 × 10^4^	Rha, Ara, Xyl, Man, Glc, Gal(0.67:53.36:17.56:0.2: 0.21:1)	3,4→)-Rhap-(1→,L-Araf-(1→,→4)-L-Arap-(1→,→3,4)-L-Arap-(1→,→2,4)-L-Arap-(1→,D-xylp-(1→,→2,3,4)-D-Xylp-(1→,→4)-DManp-1→,→2,3→)-Glcp-(1→,D-Galp-(1→,→6)-DGalp-(1→	Hao et al., (2016)

Note: rhamnose-Rha.; arabinose-Ara.; glucose-Glc.; galactose-Gla.; xylose-Xyl.; mannose-Man.; glucuronic acid-Glc A.; galactose acid-Gla A.

At present, several reports focused on the immunomodulatory activity of total polysaccharides (crude APSs) (Li, 2017; Wang et al., 2018). Although these reports are in line with the idea of “integration” of traditional Chinese medicine, they did not accurately establish the relationship between immune activity and relative Mw and elucidate the mechanism by which APSs exert immune activity at the molecular level. This research gap may be attributed to the fact that the relative Mw of crude APSs is widely distributed and an in-depth study of their immune activity and mechanisms is limited. At present, few reports focused on the structure and immune activity of APSs based on Mw distribution.

In addition to the complex chemical structure of APSs, their various biological activities, including immunomodulatory, anti-inflammatory, antioxidant, anti-tumor, and hypoglycemic, have also received extensive attention (Wang et al., 2018; Han et al., 2019; Zheng et al., 2020), as shown in Table 2. APSs play an immunoregulatory role in many aspects. In innate immunity, APSs stimulate immune cells, such as phagocytes, natural killer cells, and dendritic cells. In adaptive immunity, T and B lymphocytes are activated to directly kill target cells or produce multiple cytokines to exert their effects. Innate immunity is often a prerequisite to adaptive immunity. The immunomodulatory effect of APS is the basis of its therapeutic effect on other diseases. Therefore, the immunomodulatory effect of APS has received increasing attention and has become a research hotspot.

**TABLE 2:** APS pharmacological activity.

Biological activity	Action mechanism	References
Immunomodulation	Promoting LPS-induced peritoneal macrophages to produce TNF-*α* (*p* < 0.05)	Xu and Chen (2005)
	Significantly enhance the ability of NK cells of S-100 tumor-bearing mice to kill target cells	Weng et al., (2003)
	Increase the ratio of CD4^+^ and CD4^+^/CD8^+^ and reduce the level of T suppressor cells (CD3^+^, CD8^+^) lymphocytes	Song et al., (2005)
	Enhance NK cell activity, and increase the levels of interleukin-2 (IL-2), IL-4, IL-10 and IFN-*γ* in serum	Yan et al., (2012)
	Increase the thymus and spleen index of mice, inhibit the expression of NF-*κ*B mRNA and IL-10 mRNA in thymus and spleen lymphocytes	Liu et al., (2004)
Anti-tumor	On the one hand, T lymphocytes can secrete cytokines to regulate tumor immunity, and on the other hand, they can directly kill tumor cells through immune memory	Wei (2014)
	Down-regulate the expression of CD40 in cells	Li et al. (2010)
Anti-virus	Increase the antibody level of HBs Ag (hepatitis B surface antigen), the proliferation activity of T cells and the activity of CTL cells, significantly increase the expression of IFN-γ of CD8^+^ T cells	Du et al., (2012)
Anti-inflammatory	Treatment of lipopolysaccharide (LPS)-induced RAW 264.7 cells can down-regulate the expression of cytokines such as IFN-*γ*, IL-1*β*, IL-22 and TNF-*α*	Liao et al., (2018)
Regulates sugar metabolism	In the pathogenesis of type 1 diabetes, CD4^+^ T lymphocytes play an important role. When specific antigens stimulate antigen-presenting cells, CD4^+^ T lymphocytes are differentiated into Th1 cells	Rubin et al., (1993)
Improves cardiovascular function	Inhibit the increase of body cardiomyocyte volume, reduce the production of TNF-*α* and IL-6, reduce the expression of atrial natriuretic peptide (ANP) mRNA, and antagonize LPS	Wang (2014)

The physiological activity of polysaccharides is related to their structure and Mw. The difference in structure and Mw may directly affect the development and utilization of the medicinal value of polysaccharides. Therefore, this study separated APS into three parts according to Mw by using ultrafiltration to clarify the material basis of immunomodulation. The structural differences of the three parts were determined on the basis of monosaccharide composition, FT-IR spectrum, linkage analysis, and nuclear magnetic resonance (NMR) analysis. The immune activity of polysaccharides with different components was determined using intrinsic immunity, humoral immunity, cellular immunity, and other related indicators and then verified by cyclophosphamide immunosuppression mouse modeling. This model and pharmacological index evaluation method are relatively mature and easy to develop, guaranteeing the screening of active polysaccharides. This study explored the differences in the structure and immunomodulatory effects of different-Mw APSs, providing a reference for elucidating the relationship between APS biological activity and Mw and a guidance for the quality control of APSs and the development of new APS products.

## Materials and Methods

### Plant Materials

Wild-simulated Astragali Radix collected from Shanxi Hunyuan (SXHY) was identified as the dried root of *A. membranaceus* (Fisch.) Bge. var. *mongholicus* (Bge.) Hsiao by Professor Qin Xuemei of Shanxi University (the harvest time is 2017, and the growth period is 5 years, 20,170,812). The specimens were stored at the Modern Research Center of Traditional Chinese Medicine of Shanxi University.

### Instruments

The following instruments were used: Huapu S6000 high-performance liquid chromatograph, Chromachem evaporative light scattering detector, AC station chromatography workstation, Huapu S6000 ultraviolet (UV) detector, METTLER TOLEDO one-thousandth analytical balance, IKA RH digital magnetic stirrer, EYELA N-1100 rotary evaporation instrument, Xiangyi TL5R centrifuge, FD-1D-80 vacuum freeze dryer (Beijing Bo Yikang Experimental Instrument Co., Ltd.), infinite M200 microplate reader, Spectrum 100 (PerkinElmer Co., Ltd. USA), cell culture incubator (Li Kang Biomedical Technology Holdings Co., Ltd.), and ultrafiltration machine (Shanghai Guxin Biotechnology Co., Ltd.).

### Reagents

Dextran standards of different molecular weights (Mw: 180, 2,700, 5,250, 9,750, 13,050, 36,800, 64,450, 135,350, 300,600, and 2,000 000 Da) were purchased from China National Institute of Pharmaceutical and Biological Products. Glucose (Glu), galacturonic acid (GalA), galactose (Gal), mannose (Man), N-acetylglucosamine (GlcNAc), fucose (Fuc), rhamnose (Rha), and arabinose (Ara) were obtained from Meilun Biotechnology Co., Ltd. (Dalian, China). Papain was acquired from Solarbio (USA). HPLC-grade acetonitrile was purchased from Merck (Darmstadt, Germany). All other reagents were of analytical grade. RAW 264.7 cells (mouse mononuclear macrophage leukemia cells) was acquired from the American Model Culture Institute. RPMI-1640 medium was supplied by Cellgro, USA. Mouse spleen lymphocytes and mouse peritoneal macrophages were provided by Wuhan Psinuo Life Technology Co., Ltd. YAC-1 cells were purchased from Solarbio (USA). Lipopolysaccharide (LPS), concanavalin (ConA), MTT, and neutral red reagent were purchased from Solarbio (USA). Enzyme-linked immunosorbent assay (ELISA) kits of Mouse Immunoglobulin G (Ig G), interleukin-2 (IL-2), interleukin-4 (IL-4), interferon-*γ* (IFN-*γ*) were obtained from Solarbio (USA). LDH substrate was prepared immediately before use.

### Sample Preparation

Dried AR was crushed into powder. About 15 g of AR powder was placed into a beaker and added with deionized water to obtain a material-to-liquid ratio of 1:20. The mixture was stirred on a magnetic stirrer and subjected to hot extraction at 90°C for 4 h. After water extraction, the sample was centrifuged, filtered, and then concentrated to 150 ml. Enzymatic hydrolysis (adding 200 U papain and reacting in a constant temperature water bath at 45°C for 6 h) combined with the trichloroacetic acid method (adding 10% trichloroacetic acid to a total volume of 200 ml, placing the reaction system in an ice bath, stirring for 15 min, and then standing still for 30 min, centrifugation at 4,000 rpm for 15 min, discard the precipitate) was performed to remove protein. Anhydrous ethanol was added to a final alcohol concentration of 90%. The precipitate was collected and lyophilized to obtain crude polysaccharide powder for use.

### Preparation of Astragalus Polysaccharide With Different Molecular Weights Values

**FIGURE 1:**
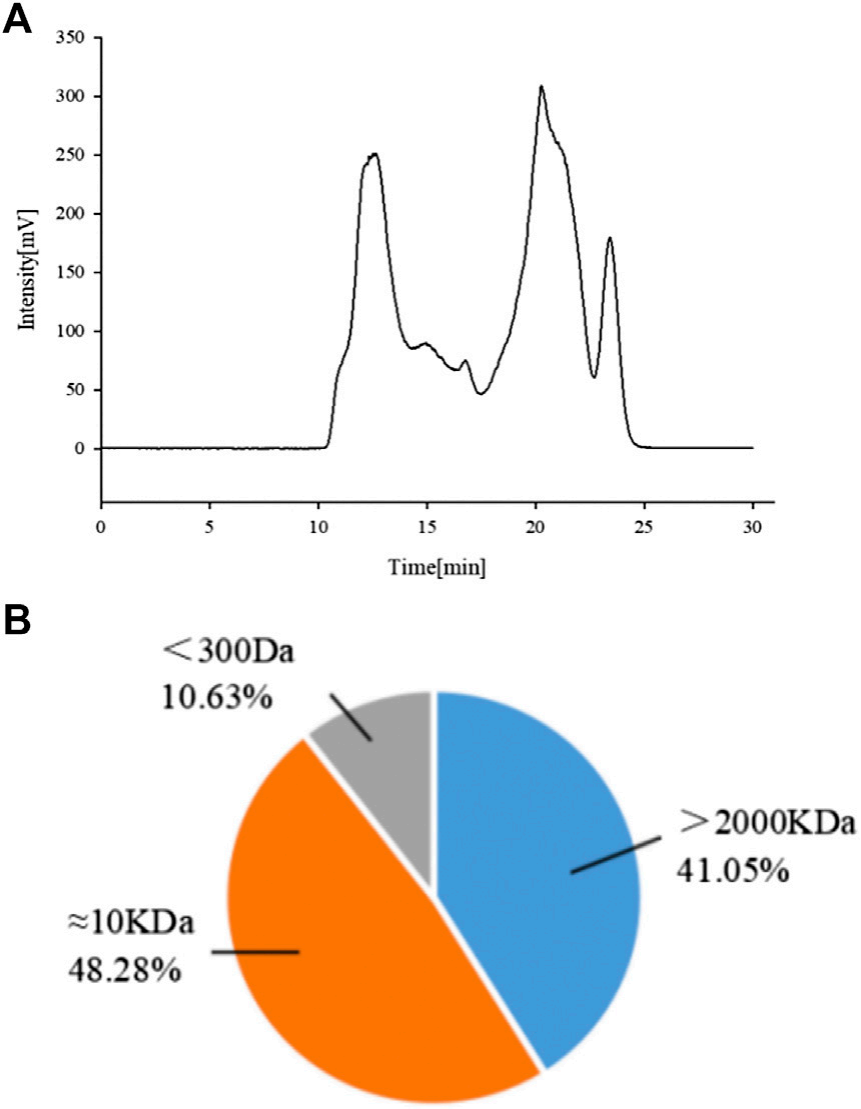
Chromatogram of total Astragalus polysaccharides (APS) via evaporative light scattering detector high-performance liquid chromatography (HPLC-ELSD) **(A)**. Molecular weight peak area of each part of APS accounts for the percentage of the total peak area **(B)**.

Previous experiments in the laboratory showed that APSs can be divided into four fractions according to the chromatogram of Mw distribution of total APS (Figure 1A). The Mw values of the first and second fractions are greater than 2,000 kDa (out of the linear range). The Mw of third fraction is about 10 kDa, and the Mw of the fourth fraction is about 300 Da (Cao et al., 2019). The APSs were formulated into a solution of 5 mg/ml and then divided into <1 kDa (APS-I), 1–30 kDa (APS-II), and >30 kDa (APS-Ⅲ) by using an ultrafiltration membrane with molecular retention of 30 and 1 kDa in the ultrafiltration system. After interception, the three parts were freeze dried separately to screen the most immunologically active fragment.

### Physicochemical Properties of Astragalus Polysaccharides With Different Molecular Weights Values

#### Determination of Molecular Weights

The relative molecular mass of APSs of different fractions was determined by high-performance gel filtration chromatography (Cao et al., 2019) with a TSK-GMPWXL gel column (10 μm, 7.8 mm × 300 mm; Tosoh, Japan) on a Huapu S6000 high-performance liquid chromatograph with an evaporative light scattering detector. Ultrapure water was used as the flow phase at a flow rate of 0.5 ml/min. The standard samples of dextrans (0.5%, w/v) were used to calibrate the standard curve. The retention time (tR) of each standard was plotted on the horizontal axis, and lgMw was plotted on the vertical axis. The relationship between lgM and tR was calculated as shown in the equation below:

$$\log M_{w}=-0.5247tR+14.763,\;R^{2}=0.9916.$$

#### Determination of Monosaccharide Composition

The monosaccharide composition of APS with different Mw values was performed by acid degradation of polysaccharides combined with pre-column derivatization. About 5 mg of the sample to be tested was placed in a hydrolysis tube, dissolved in 2 M trifluoroacetic acid, and then placed in an oven at 120°C for 2 h. A small amount of methanol was repeatedly added under reduced pressure to remove residual trichloroacetic acid. Then, the polysaccharide sample was derivatized. About 0.2 ml of the solution was mixed with PMP and NaOH solution. The solution was placed in a constant temperature metal bath and reacted for 70 min. Exactly 0.2 ml of 0.3 M HCl was added for neutralization. About 1 ml of chloroform was added for extraction, which was repeated three times to obtain an upper layer of water. Eight monosaccharide standards (Man, Rha, Fuc, Gal A, Glu, Gal, Ara, and GlcNAc) were prepared in accordance with the “sample derivatization” method.

The derivatives were analyzed on a Huapu S6000 high-performance liquid chromatograph with a UV detector by a Huapu Unitary C18 column (250 mm × 4.6 mm, 5 μm, Huapu Company). The column was eluted with potassium dihydrogen phosphate buffer (pH 6.7, 50 mM) and acetonitrile (83:17, v/v) at a flow rate of 1.0 ml/min. The detection wavelength was 250 nm.

The column was eluted with potassium dihydrogen phosphate buffer (pH 6.7, 50 mM) and acetonitrile (83:17, v/v) at a flow rate of 1 ml/min. The column temperature was set at 35°C, the chromatograms were monitored at 250 nm, and the sample injection volume was 20 µL.

#### FT-IR Analysis

APSs of different Mw values (2 mg) were mixed with KBr powder (100 mg) and pressed into pellets for FT-IR spectroscopy within 4,000–450 cm^−1^. The FT-IR spectra were recorded on an FT-IR spectrometer.

#### Methylation Analysis

Preparation of APS solution and NaH-dimethyl sulfoxide suspension: 5 mg of APS samples were obtained, added with 2 ml of anhydrous DMSO, and then magnetically stirred overnight. The fully dried NaH was quickly added to anhydrous DMSO, and the tube was magnetically stirred for 1 h to prepare a homogeneous suspension. The hydrolysis tube was always filled with N_2_ to create an oxygen-free environment.

Methylation: NaH-DMSO suspension was added to the dissolved APS samples, sealed with N_2_, ultrasonically reacted for 1 h, and then ultrasonically reacted with 1 ml of methyl iodide for 1 h. The reaction was repeated three times, and 5 ml of water was added to stop the reaction. The samples were extracted with 5 ml of CHCl_3_ four times, and the upper layer was discarded. Then, the samples were extracted with 3 ml of water four times, and the upper layer was discarded. The product was added with anhydrous Na_2_SO_4_ and filtered, and then the filtrate was dried with N_2_. The methylation product was detected by infrared spectroscopy. No absorption peak was detected at 3,400 cm^−1^, indicating the completion of the methylation reaction.

#### Hydrolysis

The methylation product was added with 2 ml of 2 M TFA to react at 120°C for 90 min, air dried, added with 10 ml of methanol, and then air dried. This procedure was repeated three times.

#### Reduction Reaction

The hydrolysate was added with 2.5 ml of 2% NaBD4 (prepared in DMSO) and reacted on a 40°C shaker for 90 min. Glacial acetic acid was slowly added until no bubbles were produced, and blow drying with air was performed.

#### Acetylation

After reduction, the product was added with 500 μL of acetic anhydride and 100 μL of 1-methylimidazole, vortexed and mixed, reacted for 15 min, and then added with 1.5 ml of water to prevent the reaction. It was then extracted with 0.5 ml of CH_2_Cl_2_ three times. The lower layers were combined, and the product was extracted with 1 ml of water. The upper layer was removed, and the product was added with anhydrous Na_2_SO_4_, filtered, dried, reconstituted with 20 μL of CH_2_Cl_2_, and then analyzed by GC-MS.

The gas chromatographic conditions were as follows: gas chromatography column, DB-5MS capillary column (30 m x 0.5 cm × 0.25 μm); carrier gas, high-purity He; injection volume, 2 μL; carrier gas flow rate, 1 ml/min; split ratio, 10:1; and inlet temperature, 220°C. The temperature programming conditions were as follows: starting temperature was 100°C and then increased to 180°C at 5°C/min for 1 min, to 190°C at 1°C/min for 2 min, to 220°C at 30°C/min for 2 min, to 230°C at 1°C/min for 2 min, and then to 280°C at 20°C/min for 10 min.

The mass spectrometric conditions were as follows: electron bombardment source; ion source temperature, 220°C; ion source, 70 eV; transmission line temperature, 250°C; scan mode for full scan (full scan); and mass scan range m/z, 30–550.

#### Nuclear Magnetic Resonance Spectroscopy

According to literature (Pu et al., 2016), exactly 30 mg of dry APS samples (APS-I, APS-Ⅱ, and APS-Ⅲ) were dissolved in 0.5 ml of D_2_O. One-dimensional 600 MHz ^1^H spectra and two-dimensional ^1^H-^13^C HSQC and HMBC were used to determine ^1^H and ^13^C chemical shifts at 30°C. The chemical shifts were referenced to the internal standard TSP with ^1^H and ^13^C at 0 ppm.

**FIGURE 2:**
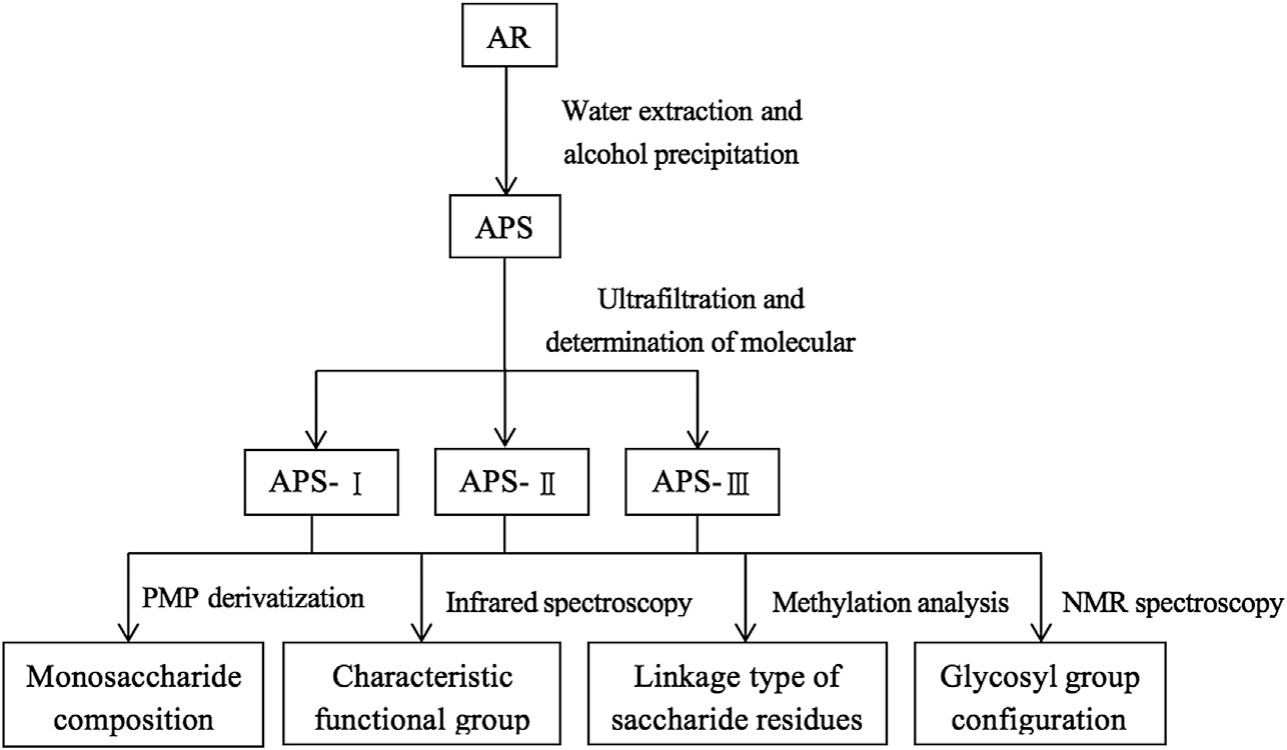
Schematic diagram of extraction and characterization of APS.

The extraction and characterization process of APS were simply represented by a schematic diagram, as shown in Figure 2.

### Screening Experiment of Cell Immunological Activity *in vitro*

#### Cell Culture

RAW264.7 cells were cultured in RPMI1640 supplemented with 10% fetal bovine serum, 100 g/L penicillin, 100 g/L streptomycin at 37°C and 5% CO_2_ saturated humidity. The experiment was carried out when the cells reached the logarithmic growth phase.

#### Polysaccharide Solution Preparation

APS solutions with different Mw values were accurately prepared with concentrations of 200, 100, 75, 50, 25, 10, and 5 μg/ml, and a microporous membrane with a diameter of 0.22 μm was used to filter and degerm the solutions for later use.

#### Spleen Lymphocyte Proliferation Assay *in vitro*

The different concentrations of polysaccharides and ConA (final concentration of 5 μg/ml) and polysaccharides and LPS (final concentration of 10 μg/ml) were added at 100 μL per well in a 96-well culture plate. Each well was then added with 100 μL/well of 1 × 10^6^ cells/mL spleen cell suspension. Six parallel wells were prepared. The plate was cultured in an incubator for 44 h. MTT (10 μL/well) was added, and the culture was continued for 4 h. After centrifugation, the supernatant was discarded in each well and added with 200 μL of DMSO. The reaction was carried out for 40 min in an incubator. Absorbance (A value) at 570 nm was detected in a microplate reader.

#### Assay of Lipopolysaccharide-Induced Spleen Lymphocyte Secretion of Immunoglobulin G *in vitro*

The different concentrations of each polysaccharide and LPS (final concentration of 10 μg/ml) were added to a 24-well culture plate at 400 μL/well. Each well was added with 1 × 10^6^ cells/mL mouse spleen lymphocyte suspension (300 μL/well) and 1 × 10^6^ cells/mL mouse peritoneal macrophage suspension (as trophoblast) at 40 μL/well. Four parallel wells were set. The culture plate was cultured in an incubator for 7 days. After centrifugation, about 50 μL of the supernatant was taken, and the IgG content was detected by ELISA. The specific operation was carried out in accordance with the instruction manual.

#### Neutral Red Uptake by Peritoneal Macrophages *in vitro*

In brief, a 1 × 10^6^ cells/mL mouse peritoneal macrophage suspension was added to a 96-well plate at 200 μL/well with four parallel wells. The culture plate was placed in an incubator for 2 h. The culture solution was discarded. Different concentrations of polysaccharides (200 μL/well) were cultured for 24 h. Each well was added with 200 μL of neutral red physiological saline solution (0.75%). After 1 h of culture, neutral red was sucked away. The residue was washed three times with PBS. Cell lysate was added at 200 μL/well and allowed to stand overnight. The absorbance value (A value) was obtained at 540 nm on a microplate reader.

#### Spleen NK Cell Activity Assay *in vitro*

In brief, mouse spleen lymphocytes were used as effector cells, and YAC-1 cells were used as target cells. Different concentrations of each polysaccharide fraction (100 μL), target cells (50 μL), and culture medium (50 μL) served as the natural release holes for target cells; different concentrations of each polysaccharide fraction (100 μL), target cells (50 μL), and 1%NP40 (50 μL) served as the maximum release holes for target cells; and different concentrations of each polysaccharide (100 μL), target cells (50 μL), and NK cells (mouse spleen lymphocyte fluid, 50 μL) served as the reaction wells. The samples were placed in each well of a 96-well culture plate, which was then incubated for 44 h. The supernatant from each well was added with 100 μL of LDH substrate. The reaction was carried out for 8 min in an incubator. Each well was added with 30 μL of 1 M HCl. Absorbance (A value) was recorded at 490 nm. The calculation formula of NK cell activity is as follows:

$$\text{NK cell activity}\left(\text{\%}\right)=\frac{{\text{OD}}_{\text{Test group}}-{\text{OD}}_{\text{natural release group}}}{{\text{OD}}_{\text{maximum release group}}-{\text{OD}}_{\text{natural release group}}}\times 100\%$$

### Screening Experiment of Cell Immunological Activity *in vivo*

#### Animal Experiment and Sample Collection

A total of 108 SPF male BALB/c mice aged 6–8 weeks and weighing 20 ± 2 g were purchased from Beijing Weitong Lihua Experimental Animal Technology Co., Ltd with animal license number SCXK (Beijing) 2016-0006. Breeding environment: temperature (25 ± 2)°C, humidity (50 ± 10)%, 12 h alternating light and dark. The experimental process is in line with the relevant regulations of the Scientific Research Ethics Review Committee of Shanxi University.

A cyclophosphamide immunosuppressive model was used. After 1 week of adaptation, the mice were divided into 12 groups (9 in each group), including a blank group (K), a model group (M), a positive drug group (total APS) (Y) (200 mg/kg/day), APS-I at low-, medium-, and high-dose groups (20, 40, and 80 mg/kg/day), APS-II at low-, medium-, and high-dose groups (25, 50, and 100 mg/kg/day), and APS-Ⅲ at low-, medium- and high-dose groups (5, 10, and 20 mg/kg/day). The dosages of APS-I, APS-II, and APS-Ⅲ were calculated based on the mass percentages of the three polysaccharides in the total polysaccharides of APS, where APS-I: APS-II: APS-Ⅲ = 40%: 50%: 10%. The high-dose groups APS-I (80 mg/kg), APS-II (100 mg/kg), and APS-Ⅲ (20 mg/kg) are equivalent to the three component doses contained in 200 mg/kg APS, and according to the high-dose group was designed for the medium and low-dose groups. Each group was intragastrically administered once daily at 0.01 ml/g body weight for 14 days. The blank and model groups contained equal volumes of water. On the ninth day after administration, except for the blank group, the other groups were intraperitoneally injected with cyclophosphamide 75 mg/kg for three consecutive days.

#### Blood Routine Testing

After the last intragastric administration, blood was taken from the eyelids and 200 μL each mouse and analyzed with a blood cell analyzer (Mairi, BC-2800 Vet).

#### Immune Organ Index

Mice were weighed 24 h after the final administration and then killed. The spleen and the thymus gland were removed and weighed. The indexes for the spleen and the thymus gland were calculated in accordance with the following formulas:

$$\text{Spleen index }=\frac{\text{Weight of spleen }\left(\text{mg}\right)}{\text{Weight of the body }\left(\text{g}\right)}$$ (1)

$$\text{Thymus index}=\frac{\text{Weight of thymus gland }\left(\text{mg}\right)}{\text{Weight of the body }\left(\text{g}\right)}$$ (2)

#### Dinitrofluorobenzene (DNFB)-Induced Delayed-type Hypersensitivity

A freshly prepared solution of 1% DNFB in acetone and sesame oil (acetone: sesame oil = 1:1) was uniformly coated on the abdomen skin of mice that had been previously shaved to sensitize. After the sensitization, cyclophosphamid was administered to induce immunosuppression on the same day. After 3 days, the right ear was evenly spread with a 1% DNFB solution to attack. After 24 h, the mice were sacrificed, and 8 mm ears were removed with a puncher and then weighed. The difference between the weight of the left and right ears was calculated as the degree of swelling.

#### Spleen Lymphocyte Proliferation Assay *in vivo*

The mouse spleen cell suspension was prepared, added to a cell culture plate, and then added with ConA and LPS respectively and incubated for 48 h. Absorbance was determined as described in Spleen Lymphocyte Proliferation Assay in vitro.

#### Determination of IL-2, IL-4, and INF-*γ* Secreted by Spleen Lymphocytes

The spleen cells and ConA were co-cultured in RPMI-1640, and the supernatant was taken. The contents of IL-2, IL-4, and IFN-*γ* were determined by ELISA in accordance with the kit instructions. The OD value of the culture solution at a wavelength of 450 nm was determined using a microplate reader. The corresponding content was determined from the cytokine standard curve.

#### Antibody Production Experiment

Spleen cell suspension and 1% sheep red blood cells (SRBCs) were added to the cell culture plates and incubated for 4 days. The tube was sequentially added with spleen cell suspension, 0.2% SRBC, and 1:10 guinea pig serum and then mixed. A blank control tube without complement was also prepared. The samples were incubated for 1 h in a 37°C water bath, removed, and then centrifuged. The OD value at 413 nm was determined with a spectrophotometer (Shimadzu UV-1201) to indicate the amount of hemoglobin.

#### Neutral Red Uptake by Peritoneal Macrophages *in vivo*

The mouse peritoneal macrophage suspension was prepared, and the absorbance was determined as described in Neutral Red Uptake by Peritoneal Macrophages in vitro.

#### Spleen NK Cell Activity Assay *in vivo*

The mouse spleen cell suspension was prepared, and the absorbance was determined as described in Spleen NK Cell Activity Assay in vitro.

#### Statistical Analysis

Data were statistically analyzed using SPSS 16.0. One-way ANOVA and *t*-test was used to determine the significance between groups, and *p* < 0.05 was considered to indicate statistical significance. All data in the tables are expressed as mean ± SD.

## Results

### Physicochemical Properties of APSs With Different Molecular Weights Values

#### Determination of Molecular Weights and Purity

**FIGURE 3:**
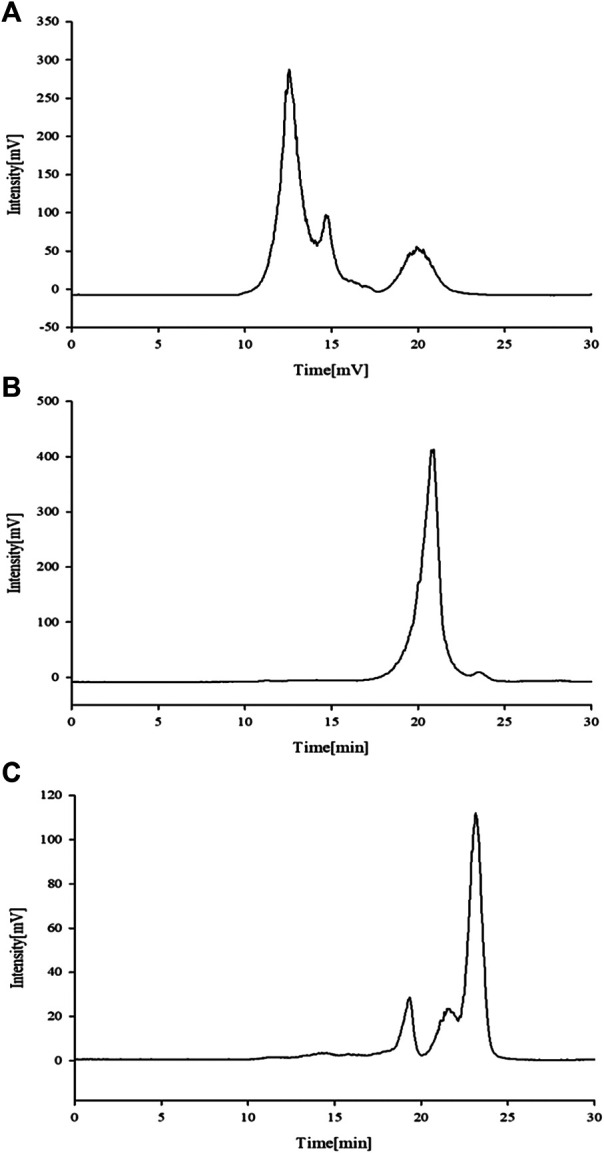
Chromatogram of APS-Ⅰ **(A)**, APS-Ⅱ **(B)** and APS-Ⅲ **(C)** by HPLC-ELSD.

A previous study obtained the chromatogram of total APS (Figure 1A). As shown in the figure, APSs can be divided into four fractions. The Mw values of the first and second fractions are greater than 2,000 kDa (out of the linear range). The Mw of the third fraction is about 10 kDa, and the Mw of the fourth fraction is about 300 Da. The molecular weight peak area of each APS fraction as a percentage of the total peak area is shown in Figure 1B. On this basis, APSs were divided into three fragments of different Mw values through ultrafiltration. The Mw distribution of the APSs with different Mw values was analyzed by high-performance gel filtration chromatography. The results are shown in Figure 3. The retention time of the three fractions of APS corresponds to the parts of total APS. The average Mw of APS-Ⅰ exceeds 2,000 kDa (more than the linear range), that of APS-Ⅱ is 1.03 x 10^4^ Da, and that of APS-Ⅲ is 272 Da. The purity of APS-Ⅰ, APS-Ⅱ, and APS-Ⅲ were 85.2%, 97%, and 84.6%, respectively. The peak area of each fraction in the chromatogram accounts for the percentage of the total peak area in Figure 3.

#### Determination of Monosaccharide Composition

**FIGURE 4:**
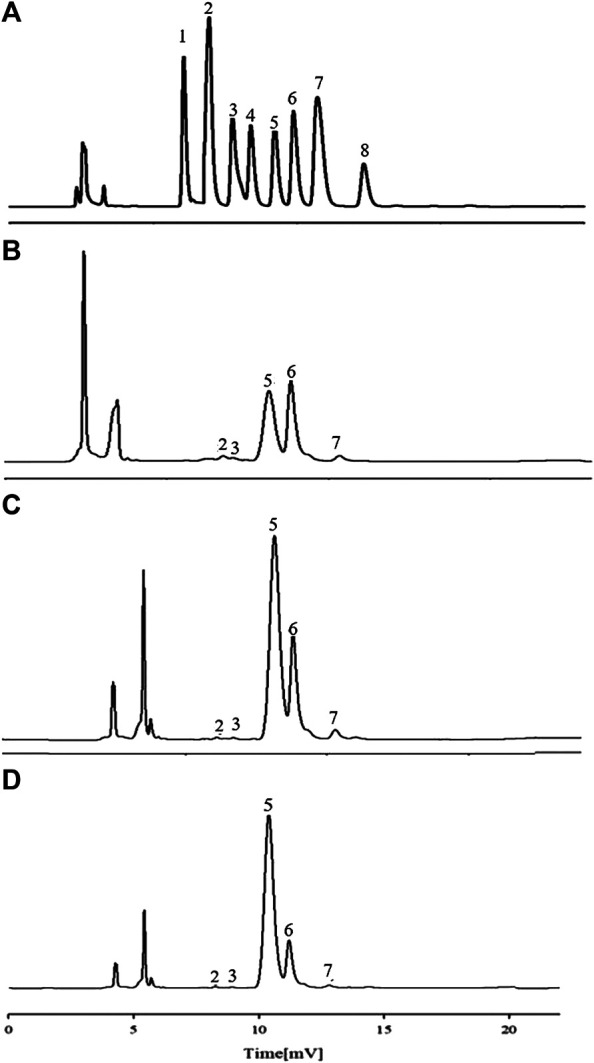
HPLC chromatograms of monosaccharide reference substances **(A)** and APS-Ⅰ **(B)**, APS-Ⅱ **(C)**, and APS-Ⅲ **(D)**.

Characteristic mapping of eight standard monosaccharide mixtures (Figure 4A) and sample AR monosaccharide (Figures 4B–D) was determined by PMP prederivatization HPLC. Comparison of the chromatograph of the monosaccharide mixed reference with the characteristic mapping of the AR monosaccharide showed that the three fractions are composed of five monosaccharides, namely, Rha, Glu, Gal, Ara, and GalA. The relative peak areas are different, indicating that the APS of the three fractions have different ratios of monosaccharide substances. The amount of Ara was 1, and the ratios of Rha, Glu, Gal, and GalA were also obtained. The ratios of Rha, Gal A, Gal, Glu, and Ara in APS-I are nearly 0.1: 0.39: 13.4: 17.2: 1; in APS-II, they are nearly 0.14: 0.14: 9.6: 24.04: 1, respectively; in APS-III, they are nearly 0.375: 0.375: 18.8: 90.5: 1. In the monosaccharide composition of three fractions, the most abundant is Glu, followed by Ara and Gal.

#### FT-IR Analysis

**FIGURE 5:**
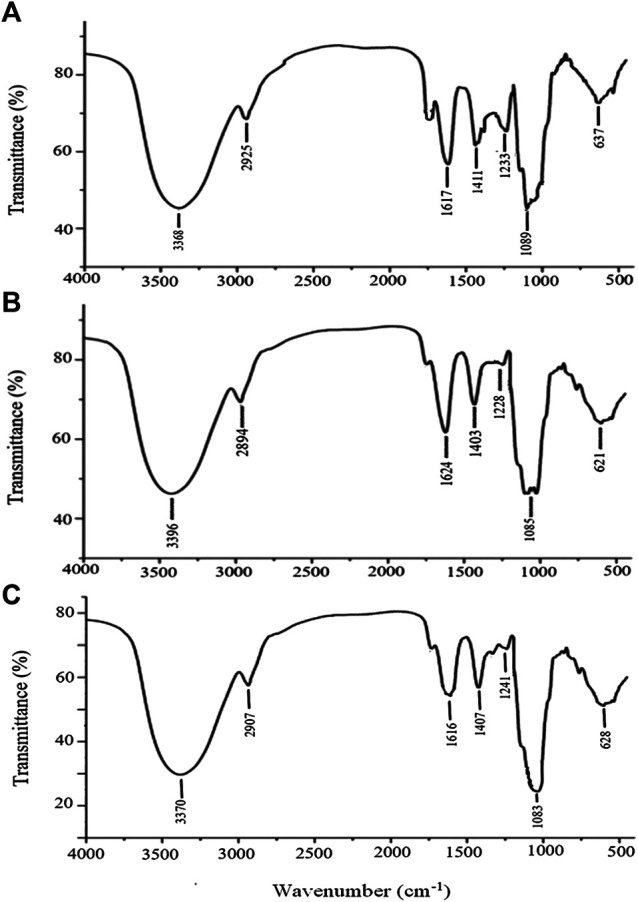
FT-IR analysis of APS-Ⅰ **(A)**, APS-Ⅱ **(B)** and APS-Ⅲ **(C)**.

The stable FT-IR technique was broadly applied to chemical bonds and structural analysis. As shown in Figure 5A, APS Ⅰ had a strong and wide band at 3,368 cm^−1^, corresponding to the O-H stretching vibration that reflected the intense inter- and intra-molecular interactions of APS. For the C-H stretching vibration, the weak absorption was at 2,936 cm^−1^. The absorption peak at about 1,629 cm^−1^ corresponded to the carboxyl group (COO^−^), which indicated that uronic acid was present in APS. The peaks at 1,411 and 1,233 cm^−1^ corresponded to the C-H and O-H variable angle vibrations, respectively. The peak at 1,089 cm^−1^ represents the vibrations of C-O-H. Similarly, the same applies to APS-Ⅱ (Figure 5B) and APS-Ⅲ (Figure 5C).

#### Methylation Analysis

**FIGURE 6:**
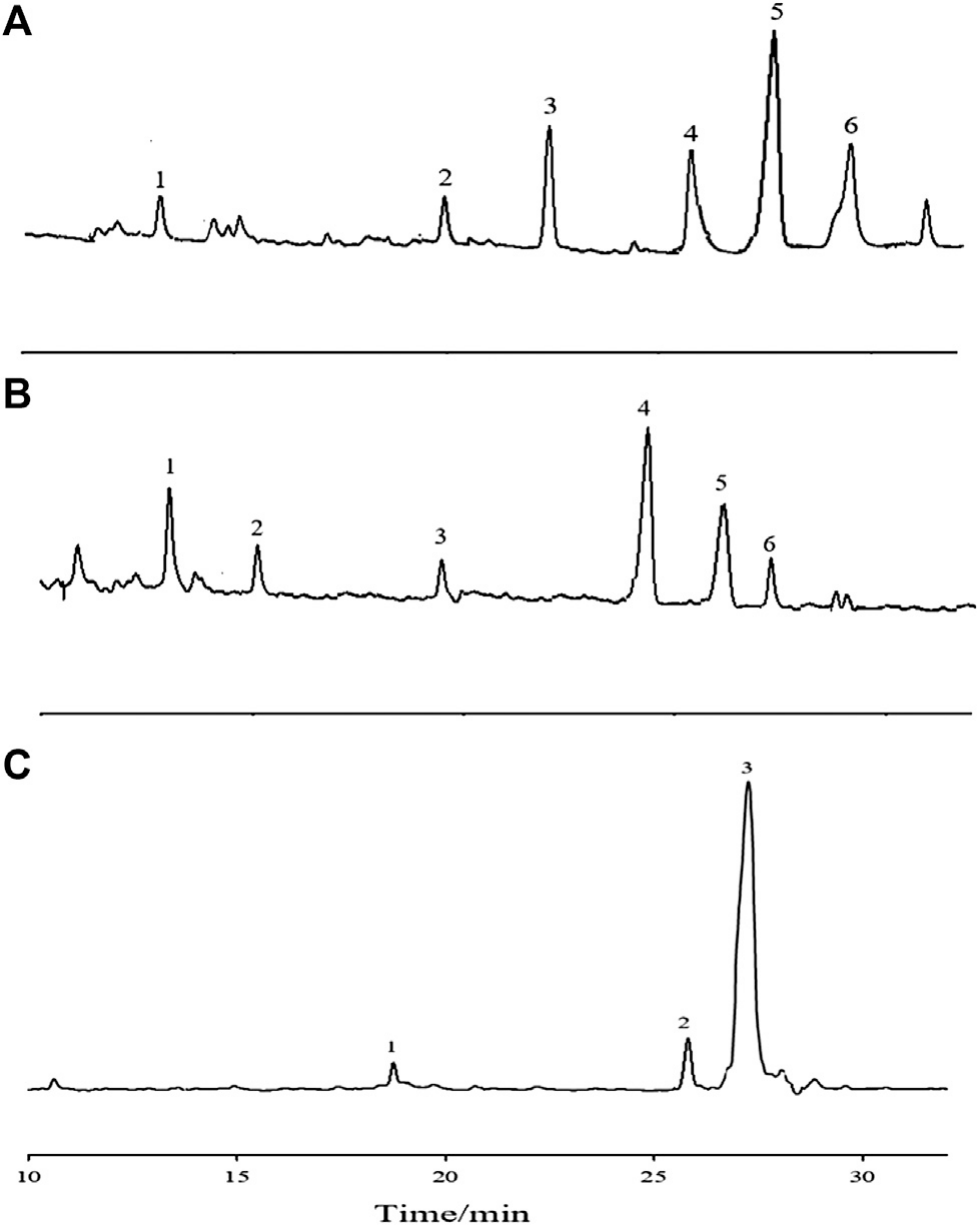
The GC-MS total-ion chromatogram of methylation analysis of APS-Ⅰ **(A)**, APS-Ⅱ **(B)** and APS-Ⅲ **(C)**.

The GC-MS total ion chromatogram of the methylation analysis of APS with different Mw values is shown in Figure 6. The mass spectrometry ion peaks of the three APS parts analyzed by GC-MS methylation were compared with the mass spectra in the database (US CCRC data). The linkage type of saccharide residues of APS-I, APS-Ⅱ, and APS-Ⅲ are summarized in Supplementary Tables S1–S3, respectively.

#### NMR Spectroscopy

**FIGURE 7:**
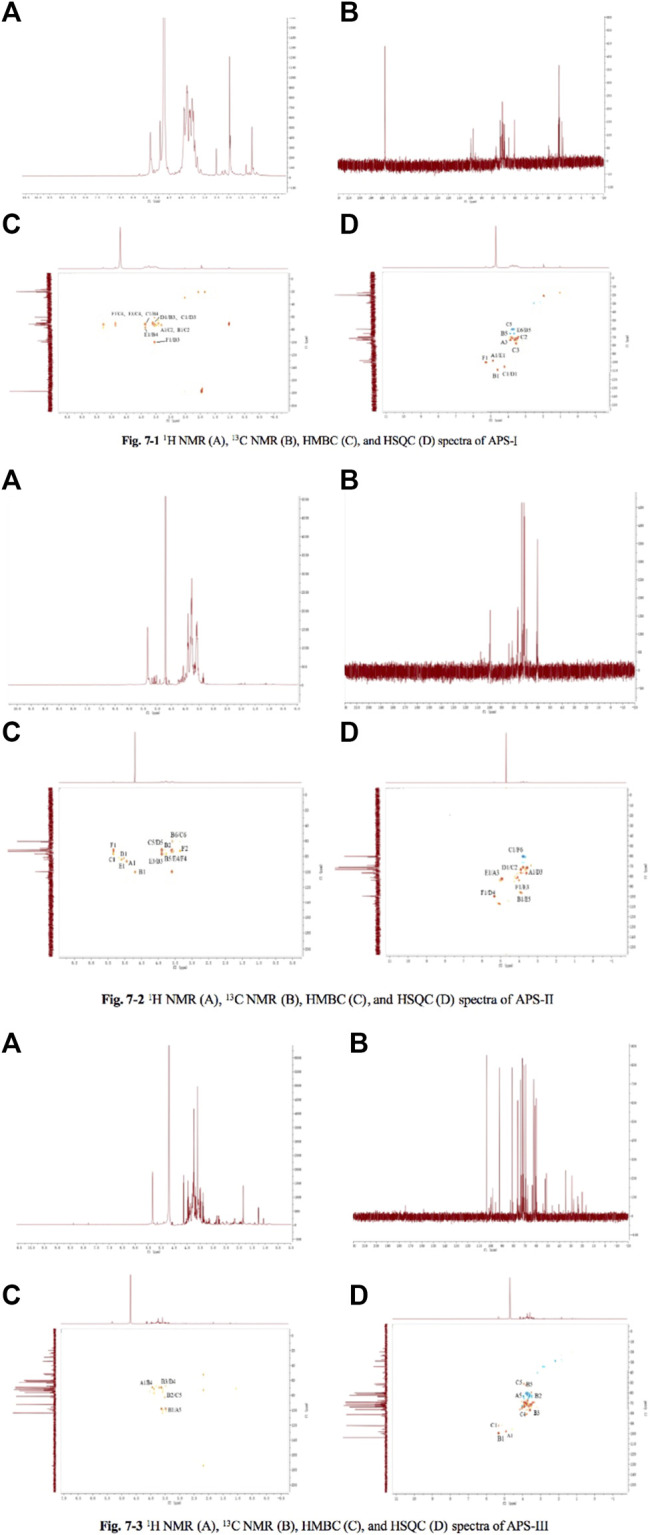
The ^1^H NMR, ^13^C NMR, HMBC, and HSQC spectra of APS-Ⅰ **(A)**, APS-Ⅱ **(B)** and APS-Ⅲ **(C)**.

On the basis of the chemical shift of the glycosyl group, the signals of APS-I, APS-Ⅱ, and APS-Ⅲ were analyzed in 1D NMR (^1^H NMR, ^13^C NMR) and 2D NMR (HSQC, HMBC), as shown in Figures 1–7. On the basis of the chemical shift of the glycosyl group in the literature, the signals were assigned and labeled separately. The resonance in the ^13^C NMR range of 90.0–112.0 ppm was attributed to the anomeric carbon atom signal, and the resonance in the ^1^H NMR range of 4.3–5.9 ppm was attributed to the anomeric hydrogen atom signal. The chemical hydrocarbon shifts of the various glycosyl groups of APS-I, APS-Ⅱ, and APS-Ⅲ are shown in Supplementary Tables S4–S6.

Methylation and NMR analyses revealed different attachment sites for monosaccharide residues. APS-I monosaccharide residue was linked to *ß*-L-Ara-(1→, →2)-*α*-D-Gal-(1→, →4)-*α*-L-Rha-(1→, →6)-*α*-D-Glu-(1→, →6)-*β*-D-Gal-(1→, →4)-*α*-D-Glu-(1→. APS-II monosaccharide residue was linked to →2,3)-*α*-L-Rha-(1→, →5)-*α*-L-Ara-(1→, →3,4)-*β*-D-Gal-(1→, →6)-*β*-D-Gal-(1→, →4)-*α*-D-Glu-(1→, →3,4,6)-*β*-D-Glu-(1→. APS-Ⅲ monosaccharide residue was linked to →5)-*α*-L-Ara-(1→, →6)-*β*-D-Gal-(1→, →4)-*α*-D-Glu-(1→).

### Effects of Astragalus Polysaccharides on Immune System *in vitro*

#### Effect of Astragalus Polysaccharides Combined With ConA on the Proliferation of Mouse Spleen Lymphocytes *in vitro*

**FIGURE 8:**
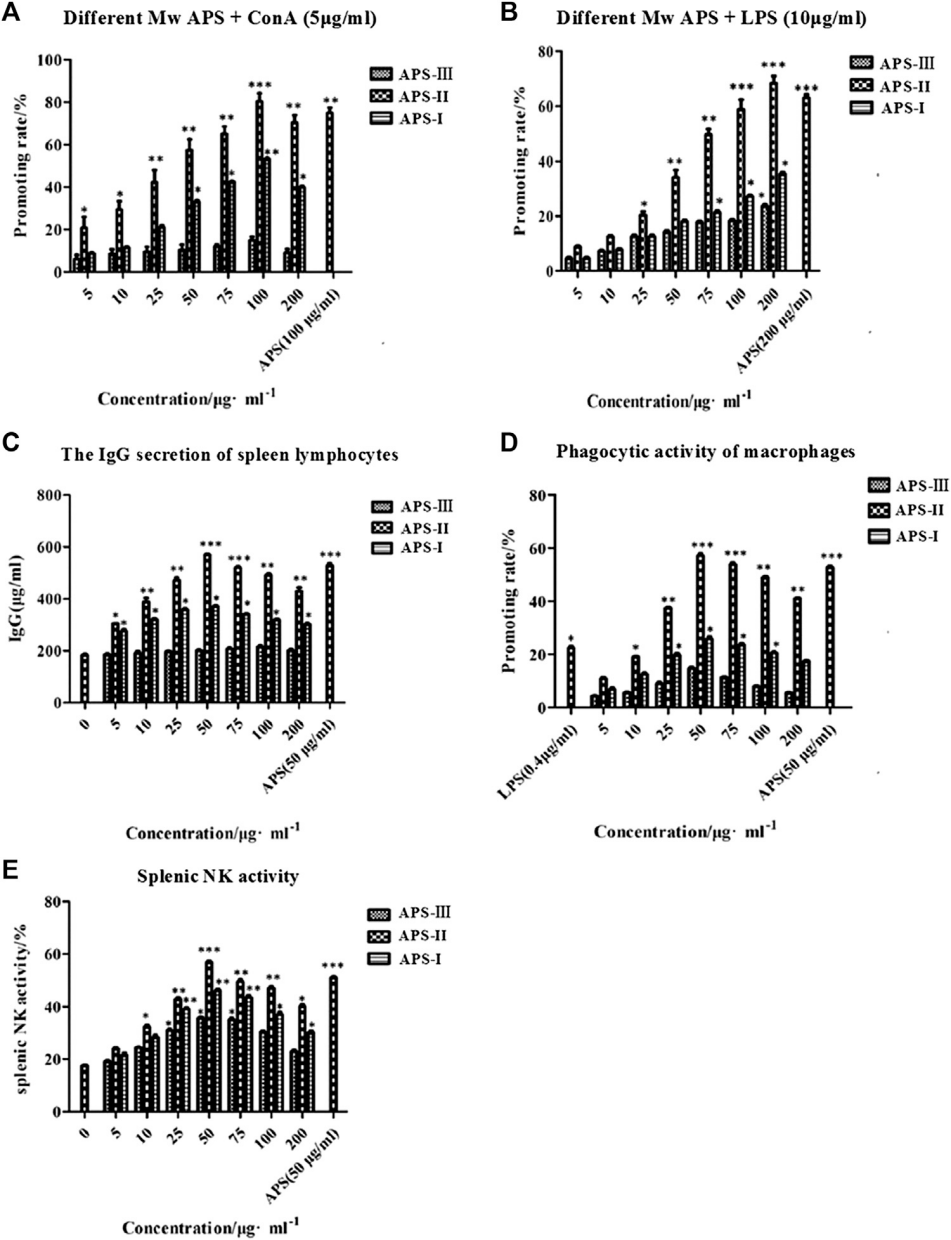
Immune-potentiating of APS on the mouse lymphocytes with ConA **(A)**, the immune-potentiating of APS on the mouse lymphocytes with LPS **(B)**, the effects of polysaccharides on the IgG secretion of spleen lymphocytes **(C)**, the functions of APS on the phagocytic activity of peritoneal macrophages **(D)**, the effects of APS on splenic NK activity **(E)**, *p < 0.05, **p < 0.01, ***p < 0.001 *vs.* the positive control. *n* = 6, *x* ± *s.*

APS is an immunomodulator that regulates the immune function of the body. ConA is a mouse T cell-specific mitogen. APSs with different Mw values promoted the proliferation of spleen lymphocytes induced by ConA to different degrees in the concentration range of 5–200 μg/ml (Figure 8A). At low concentrations, the number of spleen lymphocytes increased with increasing concentrations of APS-Ⅰ and APS-Ⅱ, and the enhancement effect reached the strongest at 100 μg/ml. The enhancement effect gradually decreased as the concentration was further increased, and the dose–effect curve was “bell-type.” The polysaccharide immune function was regulated in two ways, and an optimal dose was present. APS-Ⅲ hardly increased with increasing concentration, and it was not statistically significant. The strength of the three parts was in the order of APS-Ⅱ > APS-Ⅰ > APS-Ⅲ. The highest proliferation rate of APS-Ⅱ reached 78.56%, which is higher than the total APS at optimal. In this experiment, different-Mw APSs and total APS can promote the proliferation of mouse spleen lymphocytes in synergy with ConA. The possible target cells are T lymphocytes, indicating that APSs can enhance cellular immune function. In addition, a significant difference was found compared with the blank group (*p* < 0.05). These results indicate that APS-Ⅱ can significantly enhance the specific immunity of the body by enhancing the ConA-induced proliferation of T lymphocytes.

#### Effect of Astragalus Polysaccharide Combined With Lipopolysaccharide on the Proliferation of Mouse Spleen Lymphocytes *in vitro*

LPS is a mouse B cell-specific mitogen. The APS fractions with different Mw values promoted the proliferation of spleen lymphocytes induced by LPS to different degrees (Figure 8B). In the concentration range 5–200 μg/ml, APS exerted a dose-dependent effect on the proliferation of spleen lymphocytes. The optimal concentration of APS-Ⅰ, APS-Ⅱ and APS-Ⅲ was 200 μg/ml. Under this condition, the cell proliferation rate was the highest, reaching 23.4%, 64.2%, and 31.8%, respectively, and a significant difference was noted compared with the blank group (*p* < 0.05). The strength of the three parts was in the order of APS-Ⅱ > APS-Ⅰ > APS-Ⅲ. In this experiment, APS can synergize with LPS to promote mouse spleen lymphocyte proliferation, indicating that it promotes B lymphocyte proliferation. These results indicate that APS-Ⅱ can significantly enhance the specific immunity of the body by enhancing the LPS-induced proliferation of B lymphocytes.

#### Effects of Astragalus Polysaccharides on the Lipopolysaccharide-Induced Immunoglobulin G Secretion of Spleen Lymphocytes *in vitro*

LPS also can cause B lymphocytes to secrete IgG. In the concentration range 5–200 μg/ml, APS-Ⅰ and APS-Ⅱ significantly promoted the LPS-induced IgG secretion of spleen lymphocytes. The dose-effect curve was “bell-type,” and the optimal concentration was 50 μg/ml (Figure 8C). The strength of the three parts was in the order of APS-Ⅱ > APS-Ⅰ > APS-Ⅲ. The highest secreted amount of APS-Ⅱ reached 572.86 μg/ml, which is higher than the total APS. APS-Ⅲ exerted minimal effect on the amount of secreted IgG and did not change with concentration. These results indicate that APS-II exerts specific immunity by enhancing the LPS-induced IgG secretion of spleen lymphocytes, which may be one of the mechanisms by which APSs exert immunity.

#### Effects of Astragalus Polysaccharides on the Phagocytic Activity of Mice Peritoneal Macrophages *in vitro*

At present, the neutral red method is commonly used for detecting Mφ phagocytosis. At the concentration range of 5–200 μg/ml, the promoting rate of all APS groups was higher than that of the control group (Figure 8D). Moreover, APSs with different Mw values at 50 μg/ml increased the macrophage uptake of neutral red more than the other concentrations. APS-II increased the neutral red uptake significantly more than LPS when the concentration was greater than 25 μg/ml. However, as the APS concentration was increased, the promoting rate decreased. This result indicates that the APSs have an optimum concentration for promoting the greatest uptake of neutral red. The Mw values of the APSs influenced their uptake ability. The strength of the three parts was in the order of APS-Ⅱ > APS-Ⅰ > APS-Ⅲ. The highest promoting rate of APS-II reached 59.82%, which is higher than the total APS. These results suggest that APS-II can exert non-specific immunity by enhancing the phagocytic activity of macrophages.

#### Effects of Astragalus Polysaccharides on the Activity of Spleen NK Cells *in vitro*

NK cells are effector cells that exert non-specific immunity. As shown in Figure 8E, the spleen NK cell activity of all APS groups was higher than that of the control group in the concentration range of 5–200 μg/ml. The dose–effect curve was “bell-type,” and the optimal concentration was 50 μg/ml. The strength of the three parts was in the order of APS-Ⅱ > APS-Ⅰ > APS-Ⅲ. The highest NK cell activity of APS-Ⅱ reached 56.85%, which is higher than the total APS. These results indicate that APS-II can exert non-specific immunity by enhancing spleen NK cell activity.

### Effects of Astragalus Polysaccharides on Immune System *in vivo*

#### Effects of Astragalus Polysaccharides on Routine Hematological Parameters in Immunosuppressed Mice

After the last intragastric administration, the blood routine test results of each group of mice are shown in Table 3. The blood routine test can confirm whether the mouse model was successfully established. Blood routine indicators include the number of white blood cells, lymphocytes (Lym), red blood cells (RBCs), hemoglobin, and platelets. As shown in Table 3, the blood routine indicator levels of the model group were significantly lower than those of the blank control group (*p* < 0.01). This finding indicates the successful establishment of the model. Compared with the model group, the positive drugs can significantly increase the level of blood routine indicators (*p* < 0.01). The strength of the three parts was in the order of APS-Ⅱ > APS-Ⅰ > APS-Ⅲ, and the blood cell level was the greatest in the medium-dose APS-Ⅱ groups and was close to that in the positive drug group. Meanwhile, APS-I and APS-Ⅲ exerted less effects on blood cell levels in mice.

**TABLE 3:** The effect of APS with different MW on routine hematological parameters in mice.

Groups	White blood cell	Lymphocyte	Red blood cell	Hemoglobin	Blood platelet
	WBC(×10^9^/L)	Lym(×10^9^/L)	RBC(×1,0^12^/L)	HGB(g/L)	PLT(×10^9^/L)
K	4.63 ± 1.56	2.07 ± 0.57	9.15 ± 1.86	147 ± 5.27	997 ± 178.63
M	1.96 ± 0.96***	0.65 ± 0.34***	8.86 ± 1.21***	122 ± 9.33**	571 ± 153.74***
Y	3.91 ± 0.42^##^	1.21 ± 0.86^##^	9.23 ± 2.37^###^	139 ± 7.29^##^	716 ± 201.13^##^
APSⅠ L	2.23 ± 0.68	0.67 ± 0.15	9.07 ± 1.92^#^	128 ± 9.26	563 ± 132.16
APSⅠ M	2.35 ± 1.21 #	0.76 ± 0.26	9.09 ± 2.05^#^	133 ± 8.27^#^	575 ± 176.25
APSⅠ H	2.39 ± 1.14^#^	0.74 ± 0.36	9.11 ± 2.38^##^	135 ± 7.13^#^	584 ± 193.46
APSⅡ L	3.54 ± 0.49^##^	1.03 ± 0.71^#^	9.10 ± 1.64^##^	135 ± 10.93^#^	643 ± 221.13^#^
APSⅡ M	3.74 ± 1.12^##^	1.14 ± 0.51^##^	9.14 ± 2.52^###^	137 ± 6.73^#^	673 ± 167.89^##^
APSⅡ H	3.86 ± 0.93^##^	1.16 ± 0.27^##^	9.18 ± 1.73^###^	142 ± 9.77^##^	692 ± 173.45^##^
APSⅢ L	1.98 ± 0.76	0.70 ± 0.38	8.97 ± 2.28	125 ± 7.02	559 ± 129.76
APSⅢ M	2.07 ± 0.53	0.69 ± 0.16	9.07 ± 1.67^#^	127 ± 6.45	569 ± 145.54
APSⅢ H	2.14 ± 0.69	0.72 ± 0.24	9.11 ± 2.48^##^	130 ± 10.97^#^	574 ± 166.26

K: Blank control group, M: Model group, Y: Positive dryg grup, APSⅠ L: Ⅰ fraction of low dose group, APS Ⅰ M: Ⅰ fraction of medium dose group, APS Ⅰ H: Ⅰ fraction of high dose group, APS Ⅱ L: Ⅱ fraction of low dose group, APS Ⅱ M: Ⅱ fraction of medium dose group, APS Ⅱ H: Ⅱ fraction of high dose group, APS Ⅲ L: Ⅲ fraction of low dose group, APS Ⅲ M: Ⅲ fraction of medium dose group, APS Ⅲ H: Ⅲ fraction of high dose group. *n* = 9, *x* , *s*. **p < 0.01, ***p < 0.001 vs. blank control group; ^#^ *p* < 0.05, ^##^ *p* < 0.01, ^###^ *p* < 0.001 vs. model group.

#### Effect of Astragalus Polysaccharides on Body Weight of Immunosuppressed Mice

**FIGURE 9:**
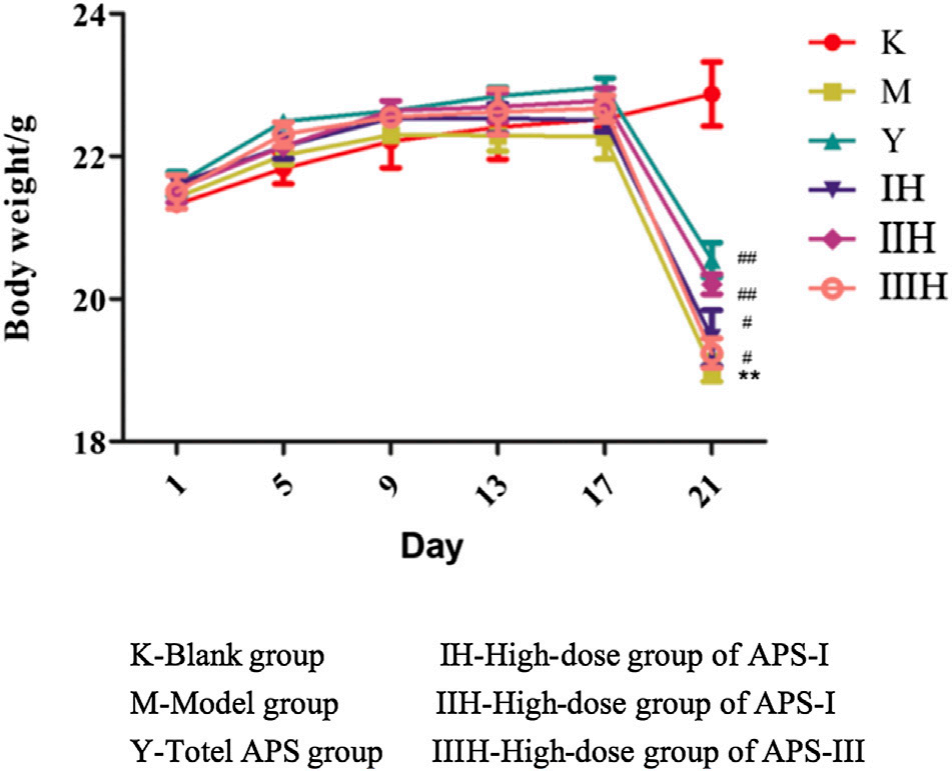
Effects of APS of different MW on the body weight of mice. (Take the high-dose group for example), *n* = 9, *x* ± *s*. **p < 0.01 vs blank control group; ^#^ *p* < 0.05, ^##^ *p* < 0.01 vs model group.

As shown in Figure 9, before the injection of cyclophosphamide, the body weight of each group increased with time, and the weight gain of the drug-administered group was higher than that of the blank control group and the model group. After the injection of cyclophosphamide, the weight of the other groups decreased except for the blank control group, but the degree of decrease of the administration groups was reduced compared with that of the model group. In the administration groups, the regulatory effect of APS-II on the body weight of immunosuppressed mice was the closest to the positive drug and was better than those of the two other fractions. The body weight of the high-dose group was higher than those of the medium- and low-dose groups.

#### Effect of Astragalus Polysaccharides on the Immune Organ Index of Mice

As shown in Table 4, the spleen and thymus indexes of the model group were significantly lower than those of the blank control group (*p* < 0.01), indicating that cyclophosphamide had a certain damage effect on the immune organs of mice. The spleen and thymus indexes of the mice increased to different degrees in the administration groups compared with the model groups, and the medium and high doses of APS-II exerted significant effects on the immune organ index (*p* < 0.05). These results indicate that APS-II can improve organ damage caused by cyclophosphamide.

**TABLE 4:** The effect of APS with different MW on immune organ index of mice.

Group	Spleen index (mg·g^−1^)	Thymus index (mg·g^−1^)
K	4.65 ± 0.22	2.91 ± 0.12
M	2.37 ± 0.12**	1.31 ± 0.05**
Y	3.35 ± 0.07^##^	1.72 ± 0.06^##^
APS Ⅰ L	2.54 ± 0.14	1.35 ± 0.03
APS Ⅰ M	2.64 ± 0.23	1.38 ± 0.11
APS Ⅰ H	2.68 ± 0.17	1.38 ± 0.03
APS Ⅱ L	2.96 ± 0.09	1.47 ± 0.07
APS Ⅱ M	3.05 ± 0.06^#^	1.53 ± 0.10^#^
APS Ⅱ H	3.08 ± 0.16^#^	1.68 ± 0.07^#^
APS Ⅲ L	2.48 ± 0.12	1.32 ± 0.06
APS Ⅲ M	2.51 ± 0.11	1.35 ± 0.09
APS Ⅲ H	2.53 ± 0.08	1.34 ± 0.10

*n* = 9, *x* , *s*. **p < 0.01 vs. blank control group; ^#^ *p* < 0.05, ^##^ *p* < 0.01 vs. model group.

#### Effect of Astragalus Polysaccharide on Delayed Hypersensitivity of Mice

Delayed hypersensitivity is a cellular immune response induced by antigens, which is related to effector T cells, phagocytic cells, and the cytokines they produce. As shown in Table 5, the mice ear swelling degree was significantly lower in the model group than in the blank control group (*p* < 0.01), indicating that cyclophosphamide can inhibit the delayed-type hypersensitivity reaction in normal mice. Compared with that in the model group, the ear swelling degree of mice in each administration group increased to different degrees, and the high dose of APS-II exerted the most significant effect (*p* < 0.01). These results indicate that APS-II can exert specific immunity by enhancing the delayed hypersensitivity reaction of mice induced by cyclophosphamide.

**TABLE 5:** The effect of APS with different MW on delayed hypersensitivity of mice.

Group	Ear swelling degree (mg)
K	7.31 ± 1.06
M	2.63 ± 0.48**
Y	5.76 ± 1.85^##^
APS Ⅰ L	3.83 ± 0.24^#^
APS Ⅰ M	4.23 ± 0.78^#^
APS Ⅰ H	4.26 ± 0.82^#^
APS Ⅱ L	4.93 ± 1.01^##^
APS Ⅱ M	5.21 ± 0.73^##^
APS Ⅱ H	5.37 ± 0.55^##^
APS Ⅲ L	3.02 ± 0.29
APS Ⅲ M	3.27 ± 0.46
APS Ⅲ H	3.31 ± 0.37

*n* = 9, *x* ± *s*. **p < 0.01 vs. blank control group; ^#^ *p* < 0.05, ^##^ *p* < 0.01 vs. model group.

#### Effect of Astragalus Polysaccharide on the Proliferation of Mouse Spleen Lymphocytes *in vivo*

**FIGURE 10:**
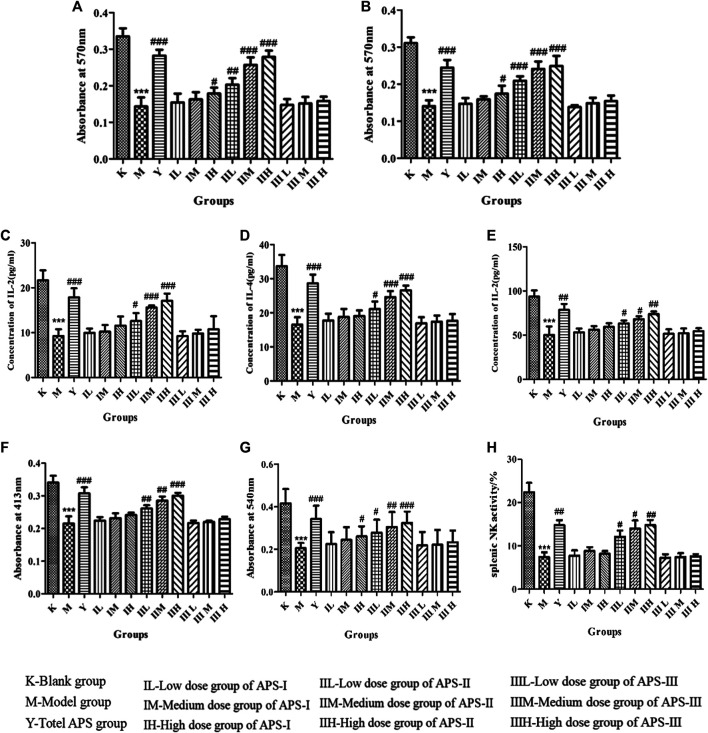
Effects of APS on proliferation of lymphocytes combined with ConA **(A)** or LPS **(B)**. Effect of APS on the secretion of IL-2 **(C)**, IL-4 **(D)**, and INF-*γ* **(E)** induction of spleen lymphocyte from immunosuppressed mice. Effect of APS on antibody production **(F)**. Effects of APS on the phagocytic activity of peritoneal macrophages **(G)**. Effects of APS on splenic NK activity (H). *n* = 6, *x* ± *s*. ***p < 0.001 vs. blank control group; ^#^ *p* < 0.05, ^##^ *p* < 0.01, ^###^ *p* < 0.001 vs. model group.

As shown in Figures 10A,B, the proliferation of mouse spleen lymphocytes *in vivo* was significantly lower in the model group than in the blank control group (*p* < 0.001), indicating that cyclophosphamide can inhibit specific immunity in normal mice. Compared with that in the model group, the proliferation of mouse spleen lymphocytes in each administration group increased to different degrees, and the high dose of APS-II exerted the most significant effect (*p* < 0.001). These results indicate that APS-II can exert specific immunity by enhancing the proliferation of mouse spleen lymphocytes.

#### Effect of Astragalus Polysaccharide on the IL-2, IL-4, and INF-*γ* Secretion of Mouse Spleen Lymphocytes

When spleen lymphocytes are activated, cytokines including IL-2, IL-4, and INF-*γ* are secreted by T lymphocytes, which are important for acute inflammatory reaction and immune enhancement. The role of IL-2 and IL-4 is to resist viral infection, activate T cells, promote B cell proliferation, and secrete antibodies. INF-*γ* can inhibit viruses, eliminate bacteria, and enhance the immune activity of macrophages and NK cells. As shown in Figures 10C–E, the cytokine level of mouse spleen lymphocytes was significantly lower in the model group than in the blank control group (*p* < 0.001). Compared with that in the model group, the cytokine level in each administration group increased to different degrees, and the high dose of APS-II exerted the most significant effect (*p* < 0.001). APS-Ⅰ and APS-Ⅲ demonstrated minimal effects on the IL-2, IL-4, and INF-*γ* secretion of mouse spleen lymphocytes. These results indicate that APS-II plays the best role among the three fractions in specific immunity.

#### Effect of Astragalus Polysaccharide on Antibody Production

B cell-mediated RBC quantitative hemolysis spectrophotometry can be used to determine the hemoglobin level released by the lysis of RBCs produced and secreted by B cells, thereby reflecting the humoral immune function of the body. As shown in Figure 10F, antibody production was significantly lower in the model group than in the blank control group (*p* < 0.001). Compared with that in the model group, the antibody production in each administration group increased to different degrees, and the high dose of APS-II exerted the most significant effect (*p* < 0.001). No significant difference was found in APS Ⅰ and APS Ⅲ.

#### Effects of Astragalus Polysaccharides on the Phagocytic Activity of Mice Peritoneal Macrophages *in vivo*

The phagocytosis of macrophages plays an important role in immunological response, which contributes to the immune function in animals. As shown in Figure 10G, phagocytosis was significantly reduced in the model group compared with the blank control group (*p* < 0.001), indicating that cyclophosphamide caused a certain damage effect on the non-specific immunity of mice. Compared with that in the model group, the phagocytosis in each administration group increased to different degrees, and the high dose of APS-II exerted the most significant effect (*p* < 0.001). Meanwhile, APS-Ⅰ and APS-Ⅲ demonstrated minimal effects on the phagocytic activity of mouse peritoneal macrophages *in vivo*.

#### Effects of Astragalus Polysaccharide on the Activity of Spleen NK Cells *in vivo*

As shown in Figure 10H, the activity of spleen NK cells in the model group was significantly lower than that in the blank control group (*p* < 0.001). Compared with the model group, the administration groups increased the activity of spleen NK cells to different degrees, and the high doses of APS-II exerted significant effects (*p* < 0.01). This result indicates that APS-II plays the best role in non-specific immunity by improving the activity of spleen NK cells.

## Discussion

### Structural Characteristics of Astragalus Polysaccharides With Different Molecular Weights Values

In this study, ultrafiltration was used for the first time to separate APS into three components, namely, APS-I, APS-II, and APS-Ⅲ. We compared the monosaccharide composition and linkage information of three fractions of different Mw values in APS and found the structural differences among the three. They have the same monosaccharide composition, but the ratios of the amounts of substances of each monosaccharide are different. They have similar functional groups as determined by IR. Methylation and NMR analyses revealed different attachment sites for monosaccharide residues. APS-I monosaccharide residue was linked to *ß*-L-Ara-(1→, →2)-*α*-D-Gal-(1→, →4)-*α*-L-Rha-(1→, →6)-*α*-D-Glu-(1→, →6)-*β*-D-Gal-(1→, →4)-*α*-D-Glu-(1→. APS-II monosaccharide residue was linked to →2,3)-*α*-L-Rha-(1→, →5)-*α*-L-Ara-(1→, →3,4)-*β*-D-Gal-(1→, →6)-*β*-D-Gal-(1→, →4)-*α*-D-Glu-(1→, →3,4,6)-*β*-D-Glu-(1→. APS-Ⅲ monosaccharide residue was linked to →5)-*α*-L-Ara-(1→, →6)-*β*-D-Gal-(1→, →4)-*α*-D-Glu-(1→. Studies have shown that 1,3-*β*-D-Glu in shiitake mushrooms and acetylated 1,4-*β*-mannan in *Aloe vera* have antitumor and immunomodulatory activities (Ming et al., 2010). 1,3-*β*-D-Man in *Tremella* can increase the leukocyte level in peripheral blood (Tian et al., 2019). 1,4-*α*-D-Glc*p* promotes T-lymphocyte proliferation (Wang et al., 2001). However, the relationship between monosaccharide residues in APS and immune activity has not been studied. Although cellular immune activity experiments *in vitro* and cyclophosphamide immunosuppression animal model experiments *in vivo* for nonspecific and specific immunoactivity indicated that the monosaccharide residues in APS include →2,3)-*α*-L-Rha-(1→, →5)-*α*-L-Ara-(1→, →3,4)-*β*-D-Gal-(1→, →6)-*β*-D-Gal-(1→, →4)-*α*-D-Glu-(1→, →3,4,6)-*β*-D-Glu-(1→), etc. may be associated with immune activity.

### Relationship Analysis of Astragalus Polysaccharides With Different Structural Characteristics and Immune Activity

APS as the most important natural effective component of the traditional Chinese medicine Astragali Radix is an important information molecule in the organism and has a strong regulatory function on non-specific immunity and specific immunity. Macrophages (Mφ) are important cellular components of the body’s immune system and play an extremely important role in the body’s innate and adaptive immune functions. It can engulf and kill pathogenic bacteria and foreign bodies, can take up and process antigens, and present antigen information to lymphocytes. The effect of polysaccharides on the function of activated NK cells is also an important aspect to evaluate their non-specific immune enhancement activities. Stimulation of macrophage and NK cells responses is one of the most important mechanisms of all known polysaccharides with immunological competence. They are the main immune cells involved in the non-specific immunity of the body. In this study, we demonstrated that APSs with different Mw values can stimulate different degree macrophages to take up neutral red and NK cell proliferation, suggesting that APS can enhance non-specific immune responses via macrophage and NK cell stimulation. The spleen is one of the most important immune organs and is the source of lymphocytes. ConA is a mouse T cell-specific mitogen. The determination of polysaccharide and ConA promotes the proliferation of mouse lymphocytes, which can reflect the effect of polysaccharide on the proliferation of T lymphocytes (Matsumoto et al., 1996). LPS is a commonly used B cell activator that causes B lymphocytes to proliferate, differentiate, and secrete IgG. APSs can synergize with LPS to promote the LPS-induced spleen lymphocyte secretion of IgG, indicating that it promotes B lymphocyte differentiation (Gordon, 1995; Zhang et al., 2003). We demonstrated that APSs of different Mw values can stimulate the specific immune system of the body by promoting lymphocyte proliferation and secreting cytokines.

The main contributor to the immunological activity of APS is APS-II (about 10 kDa). The Mw of APS-Ⅰ is greater than 2 MDa, and that of APS-Ⅲ is about 300 Da. APS-Ⅲ exerted no significant effect on enhancing the immune function of the body possibly because it is mostly monosaccharide or disaccharide, indicating that monosaccharide or disaccharide alone has no significant effect on immune cells. The activity of APS-Ⅰ was lower than that of APS-II, indicating that low-Mw APSs exert better immunomodulatory effects than high-Mw APS. Although macromolecular APSs have certain biological activities, their relative molecular mass is large, its solubility is poor, and its bioavailability is low, restricting their efficacy. APS-II with a Mw of 10 kDa can stimulate the immune system the most. It can enhance both specific and non-specific immunity in the body. Low-Mw polysaccharides have better water solubility than high-Mw polysaccharides, and the more chain breaks, the higher the water solubility. The *in vivo* animal immune activity was verified, and results showed that APS-II exerted the most obvious effect on improving the body weight and immune organ weight of cyclophosphamide-immunosuppressed mice. Among the APS fractions, APS-II showed the greatest ability to increase its specific and non-specific immunity. This result validated the results of *in vitro* cell viability screening experiments. The biological activity of APSs is related to their relative molecular mass and structural characteristics. In this study, the molecular weight of APS-Ⅱ is about 10 kDa, the polysaccharide backbone is →4)-*α*-D-Glu-(1→, and it has the best immunological activity among the APS fractions. Thus, it plays a major role in the immunological activity of the total polysaccharide APS. Deng et al. (2018) compared the antioxidant and antitumor activities of APS and their oxidative degradation fragments and found that the antioxidant and anti-tumor effects *in vitro* increase with decreasing relative molecular mass of APS degradation fragments. Zhang et al. (2015) reported that polysaccharides from *Glycyrrhiza uralensis* with low Mw exhibit higher antioxidant activities at the same concentration. Sun et al. (2012) also reported that polysaccharides isolated from *Porphyridium cruentum* with low-molecular-weight (6.53 kDa) fragments exert the strongest immunoenhancing activity. Wang et al. (2018) studied the antioxidant capacity of different Mw values of APS and showed that only APS with medium Mw and main chain *a*-D-(1, 4)-glucan display the greatest antioxidant activity and the ability to repair human renal epithelial (HK-2) cells, whereas very high or very low Mw is not conducive to the activity of APS, consistent with our results.

## Conclusion

Saccharides are the most abundant substance with the strongest immunological activity in Astragali Radix. However, systematic structure study and immunoactivity screening for different Mw polysaccharides in AR are lacking. In this study, APSs were divided into three fragments of different Mw values, >2,000 kDa (APS-Ⅰ), about 10 kDa (APS-Ⅱ), and about 300 Da (APS-Ⅲ), by using ultrafiltration for the first time. Firstly, the structural differences between three fractions compared by measuring the monosaccharide composition, FT-IR spectrum, linkage analysis and NMR analysis. Structural identification revealed that the three fractions are composed of five monosaccharides, namely, Rha, Glu, Gal, Ara, and GalA, however, they have different ratios of monosaccharide substances. And the three fractions have different attachment sites for monosaccharide residues. APS-I monosaccharide residue was linked to *ß*-L-Ara-(1→, →2)-*α*-D-Gal-(1→, →4)-*α*-L-Rha-(1→, →6)-*α*-D-Glu-(1→, →6)-*β*-D-Gal-(1→, →4)-*α*-D-Glu-(1→. APS-II monosaccharide residue was linked to →2,3)-*α*-L-Rha-(1→, →5)-*α*-L-Ara-(1→, →3,4)-*β*-D-Gal-(1→, →6)-*β*-D-Gal-(1→, →4)-*α*-D-Glu-(1→, →3,4,6)-*β*-D-Glu-(1→. APS-Ⅲ monosaccharide residue was linked to →5)-*α*-L-Ara-(1→, →6)-*β*-D-Gal-(1→, →4)-*α*-D-Glu-(1→.

Secondly cellular immune activity experiments *in vitro* and cyclophosphamide immunosuppression animal model experiments *in vivo* for nonspecific and specific immunoactivity screening methods were applied to find the most immunogenic fragment in APS. We experimentally proved that APS-II has the strongest ability to promote the phagocytosis of Raw 264.7 macrophages, enhance the killing activity of NK cells, promote the proliferation of T and B lymphocytes, and promote the production of immunoglobulin G (Ig-G) by B lymphocytes. *In vivo* experiments showed that APS-II can improve the leukopenia caused by cyclophosphamide and increase the IL-2, IL-4, and INF-γ levels in mice sera.

The immune activity of APS is related to its Mw and structure. APS-II shows stronger immunomodulatory activity than APS-I and APS-III, which may be related to its Mw, branching degree and *a*-1, four and *a*-1, six glycosidic bonds in its structure. APS-II with a low-molecular-weight is more soluble in water and absorbed by the body, so it is easier to combine with immune cell membrane receptors to exert stronger immune activity.

This research may serve as a reference for further study on APSs with different structures and immune activities, and as a guidance for the quality control of APSs and the development of new APS products. We will clarify the specific reasons for the difference in immune activity caused by structural difference in our future experiments.

## Data Availability Statement

The raw data supporting the conclusion of this article will be made available by the authors, without undue reservation, to any qualified researcher.

## Ethics Statement

The animal study was reviewed and approved by Modern Research Center for Traditional Chinese Medicine Shanxi University.

## Author Contributions

KL and Y-XC contributed equally to this work. KL, S-MJ and Y-GD provided the concept and designed the study. Y-XC conducted the analyses and wrote the manuscript. Y-XC and KL participated in data analysis. X-MQ and G-HD, provided oversight. KL contributed to revising and proof-reading the manuscript. All authors read and approved the final manuscript.

## Conflict of Interest

The authors declare that the research was conducted in the absence of any commercial or financial relationships that could be construed as a potential conflict of interest.
