# Supplementary material for: Structural Characterization and Immune Activity Screening of Polysaccharides With Different Molecular Weights From Astragali Radix
Source: Front Pharmacol. 2020 Nov 24;11:582091. doi: 10.3389/fphar.2020.582091 (PMC7774520; doi:10.3389/fphar.2020.582091)
Supplement: Supplementary file 1 [file datasheet1.docx]

**Supplementary Information**

Ke Li^1, 2^ †, Yu-xin Cao^1^†, Si-ming Jiao^2^, Yu-guang Du^2^, Guan-hua Du^3^, Yu-guang Du^2^*****, Xue-mei Qin^1^*****

^1^ Modern Research Center for Traditional Chinese Medicine, Shanxi University, Taiyuan, China, ^2^ Institute of Process Engineering, Chinese Academy of Sciences, Beijing, China, ^3^ Institute of Materia Medica, Chinese Academy of Medical Sciences & Peking Union Medical College, Beijing, China

†*These authors contributed equally to this work.*

*** Correspondence:** Xue-mei Qin (Deputy Director of Key Laboratory of Ministry)

Tel: 0086-351-701-9297

Fax: 0086-351-701-9297

Email: [qinxm@sxu.edu.cn](mailto:qinxm@sxu.edu.cn)

Yu-guang Du

Email: [yhdu@ipe.ac.cn](mailto:yhdu@ipe.ac.cn)

**Table S1** Methylation conclusion of APS-Ⅰ

| Number | t_R_/min | Suger | Methylated suger | Mass fragment  m/z | Molar ratio | Linkages type |
| --- | --- | --- | --- | --- | --- | --- |
| 1 | 12.87 | Ara- | 2,3,5-Me_3_-Ara | 43,77,87,119,  129,161,189 | 5.66 | L-Ara-(1→ |
| 2 | 15.46 | Gal- | 3,4,6-Me_3_-Gal | 43,87,101,118,129,162,189 | 3.21 | →2)-D-Gal-(1→ |
| 3 | 19.91 | Rha- | 2,3-Me_2_-Rha | 43,87,102,118,129,159,205,  233 | 2.78 | →4)-L-Rha-(1→ |
| 4 | 24.87 | Glu- | 2,3,4-Me_3_-Glu | 43,57,87,98,  116,129,158,  233 | 4.45 | →6)-D-Glu-(1→ |
| 5 | 26.43 | Gal- | 2,3,4-Me_3_-Gal | 43,71,87,102,  118,129,162,  189,233 | 7.62 | →6)-D-Gal-(1→ |
| 6 | 27.25 | Glu- | 2,3,6-Me_3_-Glu | 43,87,101,117,  129,189 | 10.37 | →4)-D-Glu-(1→ |

**Table S2** Methylation conclusion of APS-Ⅱ

| Number | t_R_/min | Suger | Methylated suger | Mass fragment  m/z | Molar ratio | Linkages type |
| --- | --- | --- | --- | --- | --- | --- |
| 1 | 13.09 | Rha- | 4-Me-Rha | 43,59,87,102,  118,129,159,  205 | 2.36 | →2,3)-L-Rha-(1→ |
| 2 | 19.84 | Ara- | 2,3-Me_2_-Ara | 59,71,87,102,  118,131,145,  162,204 | 3.08 | →5)-L-Ara-(1→ |
| 3 | 22.74 | Gal- | 2,6-Me_2_-Gal | 59,71,87,102,  118,129,145,  162,205 | 5.77 | →3,4)-D-Gal-(1→ |
| 4 | 26.63 | Gal- | 2,3,4-Me_3_-Gal | 59,71,87,102,  118,129,162,  189,233 | 4.42 | →6)-D-Gal-(1→ |
| 5 | 27.51 | Glu- | 2,3,6-Me_3_-Glu | 43,59,87,101,  117,129,189 | 14.56 | →4)-D-Glu-(1→ |
| 6 | 30.7 | Glu- | 4-Me-Glu | 59.85,102,118,127,142,201,  261 | 6.23 | →3,4,6)-D-Glu-(1→ |

**Table S3** Methylation conclusion of APS-Ⅲ

| Number | t_R_/min | Suger | Methylated suger | Mass fragment  m/z | Molar ratio | Linkages type |
| --- | --- | --- | --- | --- | --- | --- |
| 1 | 19.17 | Ara- | 2,3-Me_2_-Ara | 59,71,87,102,  118,131,145,  162,205 | 1.07 | →5)-L-Ara-(1→ |
| 2 | 26.34 | Gal- | 2,3,4-Me_3_-Gal | 59,71,87,102,  118,129,162,  189,233 | 4.58 | →6)-D-Gal-(1→ |
| 3 | 27.35 | Glu- | 2,3,6-Me_3_-Glu | 43,59,87,101,  118,129,189 | 26.94 | →4)-D-Glu-(1→ |

**Table S4** ^1^H and ^13^C chemical shift assignments δ (ppm) of APS-Ⅰ

| Glycosyl residues | Chemical shifts, δ(ppm) | | | | | |
| --- | --- | --- | --- | --- | --- | --- |
|  | H1/C1 | H2/C2 | H3/C3 | H4/C4 | H5/C5 | H6/C6 |
| β-L-Ara-(1→ (A) | 4.47/102.2 | 4.32/83.9 | 3.79/76.8 | 3.65/70.1 | 3.72/73.7 | -/- |
| →2)-α-D-Gal-(1→ (B) | 5.28/99.7 | 4.09/79.7 | 3.79/73.8 | 3.64/70.3 | 3.57/66.2 | 3.63/67.4 |
| →4)-α-L-Rha-(1→ (C) | 5.12/97.4 | 4.41/71.6 | 3.99/73.5 | 3.52/68.6 | 3.78/63.3 | 1.64/17.4 |
| →6)-α-D-Glu-(1→ (D) | 4.94/97.6 | 3.75/70.8 | 3.58/72.1 | 3.61/75.4 | 3.86/67.2 | 3.64/69.7 |
| →6)-β-D-Gal-(1→ (E) | 4.81/101.7 | 4.09/84.1 | 3.58/74.7 | 3.84/77.5 | 3.67/69.6 | 3.83/70.2 |
| →4)-α-D-Glu-(1→ (F) | 4.98/96.8 | 3.54/73.4 | 3.62/77.1 | 3.47/73.5 | 3.53/69.6 | 3.35/65.7 |

**Table S5** ^1^H and ^13^C chemical shift assignments δ (ppm) of APS-Ⅱ

| Glycosyl residues | Chemical shifts, δ(ppm) | | | | | |
| --- | --- | --- | --- | --- | --- | --- |
|  | H1/C1 | H2/C2 | H3/C3 | H4/C4 | H5/C5 | H6/C6 |
| →2,3)-α-L-Rha-(1→ (A) | 5.13/98.4 | 4.23/79.3 | 4.01/76.9 | 3.62/71.3 | 3.78/69.4 | 1.18/21.6 |
| →5)-α-L-Ara-(1→ (B) | 5.17/102.7 | 4.12/81.8 | 3.86/74.2 | 3.99/80.5 | 3.79/66.7 | -/- |
| →3,4)-β-D-Gal-(1→ (C) | 4.73/103.1 | 3.91/82.3 | 3.79/80.2 | 3.84/74.4 | 3.59/72.0 | 3.67/58.3 |
| →6)-β-D-Gal-(1→ (D) | 4.84/104.6 | 4.04/83.7 | 3.58/72.8 | 3.69/77.3 | 3.80/67.5 | 3.74/70.1 |
| →4)-α-D-Glu-(1→ (E) | 5.00/98.7 | 3.56/74.6 | 3.78/77.4 | 3.54/71.5 | 3.71/69.2 | 3.64/67.4 |
| →3,4,6)-β-D-Glu-(1→ (F) | 5.35/102.8 | 4.10/87.1 | 3.82/73.1 | 3.86/73.8 | 3.65/78.9 | 3.59/72.3 |

**Table S6** ^1^H and ^13^C chemical shift assignments δ (ppm) of APS-Ⅲ

| Glycosyl residues | Chemical shifts, δ(ppm) | | | | | |
| --- | --- | --- | --- | --- | --- | --- |
|  | H1/C1 | H2/C2 | H3/C3 | H4/C4 | H5/C5 | H6/C6 |
| →5)-α-L-Ara-(1→ (A) | 5.28/101.6 | 4.23/80.4 | 3.81/74.8 | 4.01/78.2 | 3.66/65.7 | -/- |
| →6)-β-D-Gal-(1→ (B) | 4.78/103.1 | 4.02/82.8 | 3.65/73.5 | 3.83/77.2 | 3.59/70.1 | 3.76/71.4 |
| →4)-α-D-Glu-(1→ (C) | 4.97/99.3 | 3.64/73.7 | 3.72/76.1 | 3.48/74.2 | 3.66/70.6 | 3.37/65.1 |

Supplementary Figure 1 Standard curve of different concentrations of glucose


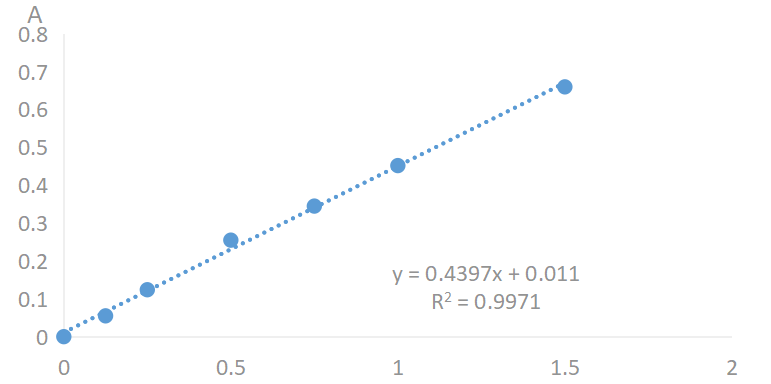

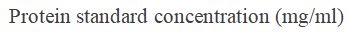


Supplementary Figure 2 Standard curve of different concentrations of protein standards
